# Neural networks improve repeatability of intravoxel incoherent motion (IVIM) parameter estimation in pancreatic diffusion-weighted MRI

**DOI:** 10.1007/s11517-026-03637-6

**Published:** 2026-07-29

**Authors:** Nitzan Avidan-Pearl, Daphna Link-Sourani, Ram Weiss, Moti Freiman

**Affiliations:** 1 Faculty of Biomedical Engineering, Technion Israel Institute of Technology, Haifa, 3200003 Israel; 2 The May-Blum-Dahl MRI Research Center, Faculty of Biomedical Engineering, Technion Israel Institute of Technology, Street, Haifa, 3200003 Israel; 3 Department of Pediatrics, Ruth Children’s Hospital, Rambam Medical Center, Haifa, Israel

**Keywords:** Intravoxel incoherent motion, Pancreas, Neural-networks, Repeatability

## Abstract

**Abstract:**

Intravoxel incoherent motion (IVIM) analysis in diffusion-weighted MRI (DWI-MRI) shows potential for characterizing pancreatic tissue, but its clinical application remains limited by sensitivity to fitting algorithms. This study assessed the repeatability of neural network (NN)-based IVIM fitting versus classical nonlinear least-squares in pancreatic DWI. The repeatability cohort included ten healthy volunteers and two type 1 diabetes (T1D) individuals, each scanned twice; the glucose-response cohort included three T1D participants and three healthy controls scanned pre- and post-oral glucose. Diffusion data were acquired at 13 b-values (0–1200 s/mm$^2$). Four NN-based methods (IVIM-NET, SUPER-IVIM-DC, U-Net, IVIM-MORPH) were compared with two classical approaches (SLS, SLS-TRF) using full (13-point) and reduced (7-point) protocols. Repeatability was quantified using within-subject coefficient of variation (wCV) and Bland-Altman analysis. All NN-based methods significantly improved test-retest repeatability of the perfusion fraction *f* compared with classical approaches (*p<0.05*), with SUPER-IVIM-DC showing the lowest wCV and IVIM-MORPH offering balanced performance across parameters. A reduced protocol shortened scan time through fewer acquisitions while maintaining or improving repeatability compared to the full protocol. Preliminary glucose-response results show both NN and classical IVIM analyses detect physiologically relevant changes. Parameter estimates varied across NN architectures, requiring further validation to establish accuracy.

**Graphical abstract:**

**Figure:**
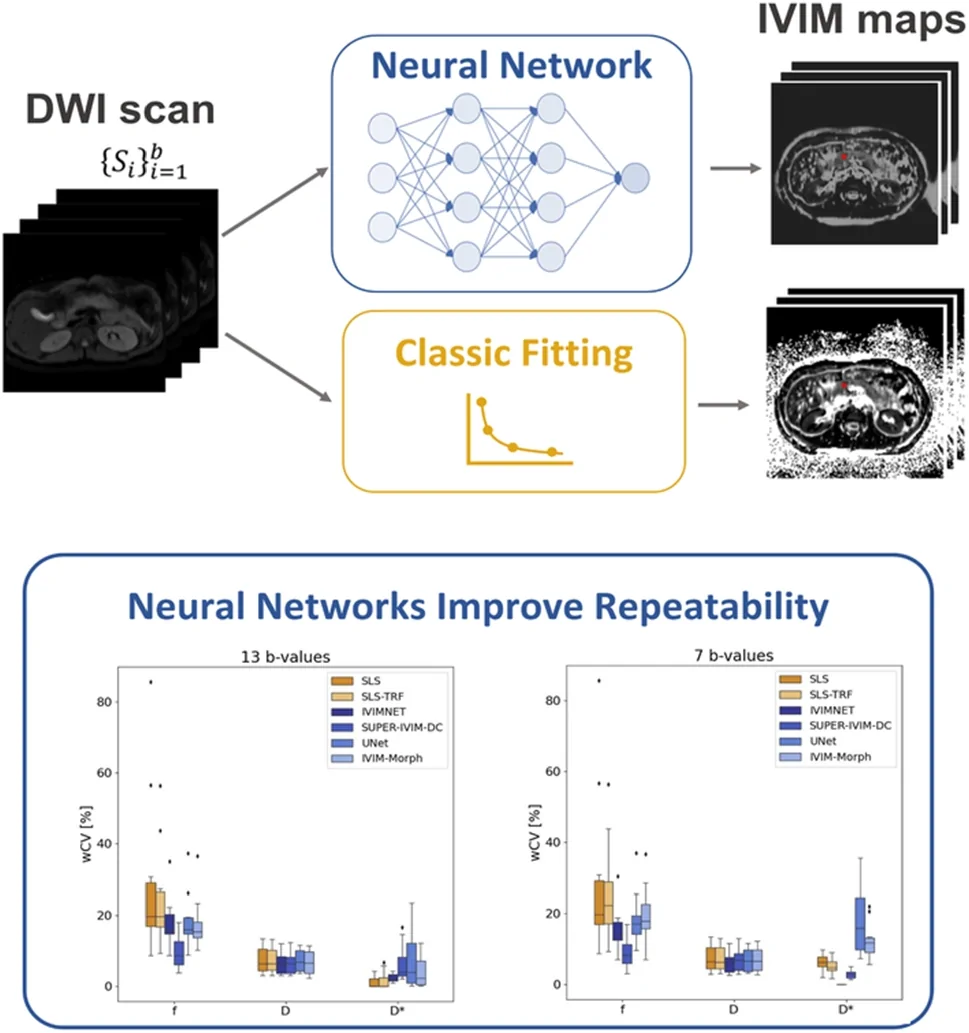

## Introduction

Intravoxel incoherent motion (IVIM) is a noninvasive MRI technique that enables simultaneous quantification of tissue diffusion and pseudo-diffusion. It is based on diffusion-weighted MRI (DWI) acquired at multiple *b*-values, where the signal attenuation is modeled using a bi-exponential function to reflect underlying microstructural and vascular properties. The DWI signal at a given *b*-value $b_i$ is described as:

1 $$\begin{aligned} S(b_i) = S_0 \left( \left( 1-f \right) e^{-b_i D} + f e^{-b_i (D+D^*)} \right) , \end{aligned}$$

where $S_0$ is the non-diffusion-weighted signal, *D* is the diffusion coefficient, $D^*$ is the pseudo-diffusion coefficient, and *f* represents the pseudo-diffusion fraction. These parameters provide insight into tissue microarchitecture and microvascular flow, supporting disease characterization across a variety of clinical contexts [1, 2].

In pancreatic imaging, IVIM-derived parameters have shown promise in several clinical applications. They have been used to differentiate benign from malignant lesions [3, 4], characterize tumor properties [5, 6], assess microvascularity [7], and predict both tumor grade and fibrosis in pancreatic ductal adenocarcinoma [8]. Beyond oncology, IVIM MRI also holds potential for investigating metabolic disorders. Recent studies suggest that IVIM may detect early *β *-cell dysfunction in asymptomatic individuals with hyperglycemia [9], indicating its promise for early detection of metabolic disease. Its parameters may further support noninvasive assessment of capillary perfusion alterations and glucose-stimulated responses, offering potential for distinguishing diabetic from non-diabetic pancreatic conditions. However, additional studies in diabetic populations are needed to establish clinical relevance [10].

Despite its clinical potential, IVIM parameter reproducibility in the healthy pancreas remains moderate, with considerable variability across scanners and acquisition protocols [11]. This highlights the need for more robust estimation techniques. Traditional voxelwise non-linear least squares (NLLS) fitting is particularly susceptible to noise, motion artifacts, and parameter initialization [12, 13]. Bayesian methods have improved estimation stability by incorporating prior information [14, 15], while recent approaches like kernelized fitting further enhance robustness through spatial regularization, using total variation penalties to enforce smoothness and suppress noise across neighboring voxels [16].

Motion artifacts remain a major challenge in abdominal IVIM imaging, particularly under free-breathing conditions. To address this, methods such as SIR-ME—which integrates image registration with model fitting [17]—and probabilistic binning strategies [18] have been proposed to enhance parameter stability by compensating for respiratory-induced signal variability.

Neural network (NN)–based approaches have recently emerged as powerful alternatives to classical IVIM fitting methods, offering improved robustness and parameter stability. Early models based on fully connected architectures [19, 20] perform voxelwise fitting, estimating parameters independently at each voxel. While effective, this approach does not leverage spatial context. Convolutional neural networks (CNNs), such as those proposed by Vasylechko et al. [21], incorporate spatial correlations by learning from local neighborhoods, producing smoother and more consistent parameter maps. More recently, Kertes et al. [22] introduced a motion-robust self-supervised CNN that explicitly addresses respiratory motion, further enhancing parameter stability under free-breathing conditions. These advances position NN-based methods as promising tools for clinically reliable IVIM analysis, particularly in anatomically and physiologically challenging regions like the pancreas.

Previous studies have demonstrated that neural network and Bayesian approaches outperform classical NLLS fitting in terms of repeatability and robustness [23, 24]. More recently, Kaandorp et al. [25] explored the impact of various training strategies on NN-based IVIM modeling in abdominal imaging. However, to date, no study has systematically evaluated and compared state-of-the-art NN architectures—particularly those incorporating motion compensation and self-supervision—in the pancreas, where respiratory motion presents persistent challenges to accurate parameter estimation.

This study aims, therefore, to systematically evaluate the performance of NN–based IVIM fitting methods in pancreatic DWI. Specifically, we:

1. Compare the repeatability and robustness of NN-based approaches with classical NLLS methods. We evaluate Segmented Least Squares (SLS)[26] and SLS with Trust Region Reflective optimization (SLS-TRF)[27] alongside four NN-based models: the voxelwise IVIM-NET [19], SUPER-IVIM-DC [20], a spatially aware self-supervised U-Net [28], and the motion-robust IVIM-MORPH [22].
2. Assess the training-to-training reproducibility of NN-based methods by evaluating their consistency across repeated training sessions.
3. Explore the sensitivity of IVIM parameters to glucose intake in a small pilot cohort of three pairs of Type 1 Diabetes (T1D) and control subjects.

By identifying computational strategies that improve the reliability of IVIM parameter estimation in the pancreas, this work aims to advance the clinical utility of quantitative DWI in abdominal imaging.

## Methods

### Participants and MRI acquisition

The institutional ethics committee approved this study, and all participants provided written informed consent. We recruited a total of 16 participants across two complementary studies. For the repeatability assessment, we enrolled twelve participants consisting of ten healthy volunteers (mean age±SD: 27±2.4 years; 4 females) and two T1D patients (both female; ages 19 and 20 years). Each participant underwent two MRI scans separated by a few-minute interval outside the scanner. For the exploratory glucose-response pilot study, we enrolled six participants, three T1D patients and three age- and sex-matched healthy controls, organized into three matched pairs (Pair 1: T1D, 28 years, male; control, 26 years, male. Pair 2: T1D, 20 years, female; control, 20 years, female. Pair 3: T1D, 21 years, female; control, 19 years, female). This cohort was formed by including the two T1D patients from the repeatability assessment and recruiting four additional participants (one T1D patient and three healthy controls). All participants in the glucose-response study underwent MRI imaging at baseline and again approximately 30 minutes after oral glucose ingestion (15 g Glucostandard, Gefen Medical LTD) to assess glucose-induced changes in IVIM parameters.

We acquired respiratory-triggered diffusion-weighted images (DWI) of the upper abdomen in the axial plane using a 3T MRI system (MAGNETOM Prisma, Siemens Healthineers, Erlangen, Germany) equipped with a 30-channel coil. The protocol included thirteen b-values: 0, 10, 20, 30, 40, 50, 60, 80, 100, 200, 400, 800, and 1200 s/mm$^2$.

Imaging was performed using a 2D echo-planar imaging (EPI) sequence with monopolar diffusion encoding applied along six approximately uniformly distributed diffusion directions. We acquired one average for b-values between 0 and 400 s/mm$^2$, two averages for 800 s/mm$^2$, and three averages for 1200 s/mm$^2$. Directionally averaged images (from the six diffusion directions) automatically generated by the scanner were used for all analyses.

Acquisition parameters were as follows: repetition time (TR), 5400 ms; echo time (TE), 49 ms; field of view, 360 *× * 360 mm$^2$; matrix size, 196 *× * 196; slice thickness, 4 mm (12 slices, 30% gap); phase encoding direction, anterior-posterior; fat suppression using spectral fat saturation; parallel imaging using GRAPPA (acceleration factor 2) with 6/8 partial Fourier; and receiver bandwidth, 1822 Hz/pixel. The reconstructed voxel size was 1.8 *× * 1.8 *× * 4.0 mm$^3$, and the average total acquisition time was 9 minutes and 7 seconds.

### Image analysis

We applied six fitting methods to the dataset to compute pixel-wise maps of IVIM model parameters. We evaluated each method using both a full set of 13 b-values and a reduced set of 7 b-values (0, 50, 100, 200, 400, 800, and 1200 s/mm$^2$). We designed the reduced b-value protocol according to prior studies [29, 30], which demonstrated that carefully optimized schemes can yield reliable IVIM parameter estimates. The b=0 image was retained for signal normalization. We selected low-to-intermediate b-values (50, 100, and 200 s/mm$^2$) to characterize the perfusion-sensitive signal decay while limiting instability associated with very low b-value acquisitions. Higher b-values (400, 800, and 1200 s/mm$^2$) were retained to characterize the diffusion-dominated regime and support robust estimation of the diffusion coefficient *D*. This reduced protocol enabled a total acquisition time of less than 5 minutes. The evaluated methods included four NN–based approaches and two classical NLLS techniques.

- **IVIM-NET**: An unsupervised, physics-informed DNN designed for IVIM parameter estimation. We used the optimized unsupervised implementation IVIM-NET$_{\textrm{optim}}$ introduced by Kaandorp et al. [19]. The model employs a fully connected architecture with parallel sub-networks to estimate *D*, $D^*$, and *f*, using scaled sigmoid activations to enforce physiologically plausible outputs. Training is performed by minimizing the reconstruction error between measured and model-predicted signals based on the IVIM equation. The architecture and training strategy were adapted to pancreas-relevant simulated data ranges.
- **SUPER-IVIM-DC** [20]: A supervised neural network optimized for reduced b-value schemes. In contrast to IVIM-NET, which is self-supervised through the forward signal model, SUPER-IVIM-DC is trained on simulated data with known IVIM parameters. The network learns a direct regression from the input signal decay to *D*, $D^*$, and *f*. In addition to the parameter-regression term, training includes a data-consistency term that penalizes disagreement between the measured input signal and the signal reconstructed from the predicted IVIM parameters. This hybrid loss was introduced to improve generalization when only a limited number of *b*-values are available. In this work, we used the original SUPER-IVIM-DC architecture and training strategy, with pancreas-relevant simulated IVIM parameter ranges used to match the target application.
- **U-Net**: The U-Net approach was a subject-specific self-supervised convolutional neural network that estimated voxel-wise *D*, $D^*$, and *f* maps directly from the subject’s diffusion-weighted image stack. Unlike the multilayer perceptron used in IVIM-NET, the U-Net exploits spatial context through an encoder-decoder architecture with convolutional filters and skip connections [28]. The predicted parameter maps were converted back to diffusion-weighted signals through the IVIM forward model, and training minimized the weighted standard error of regression (WSER) loss [22] between measured and reconstructed signals. Training was performed separately for each subject (batch size *=1*), using Adam optimization with an initial learning rate of $5\times 10^{-4}$, a reduce-on-plateau scheduler, and early stopping after 10 non-improving iterations, with a maximum of 2500 iterations.
- **IVIM-MORPH** [22]: A subject-specific self-supervised extension of the U-Net that incorporates motion correction. The method consists of two coupled modules: (i) a quantitative IVIM module that estimates *D*, $D^*$, and *f*, and (ii) a VoxelMorph-based registration module for non-rigid alignment of diffusion-weighted images acquired at different *b*-values [31]. This joint framework addresses respiratory and bulk motion in abdominal DWI, which can violate the voxel-wise correspondence assumed by standard IVIM fitting. By combining deformable registration with IVIM parameter estimation, IVIM-MORPH aims to produce motion-corrected images and more stable parameter maps. In this study, training followed the subject-specific U-Net strategy, with pancreas-adapted parameter bounds and learning-rate adjustments to improve convergence.

The remaining two methods were conventional segmented fitting approaches for estimating IVIM parameters from the bi-exponential signal model. They were implemented as benchmark analyses, following recent IVIM consensus recommendations for a predefined linearized segmented fit to improve reproducibility and cross-study comparison [32]. The OSIPI IVIM-MRI CodeCollection was used as a reference implementation for the standardized fitting framework [33].

- **SLS**: The standardized segmented least-squares fit was used as the primary benchmark method. First, the high-*b*-value signal decay was fitted with a linearized mono-exponential model, using $b>200~\mathrm {s/mm^2}$, where the pseudo-diffusion contribution is assumed to be negligible. This step estimates *D* from the slope of the log-transformed signal and estimates *f* from the intercept. In the second step, the full bi-exponential IVIM model was fitted to the measured signal using the segmented estimates as initialization, allowing estimation of $D^*$ [26].
- **SLS-TRF**: SLS-TRF used the same segmented initialization as SLS, but the final nonlinear fitting step was performed with the trust-region reflective algorithm [27]. This optimizer allows explicit lower and upper bounds on *D*, $D^*$, and *f*, reducing non-physiological estimates and improving convergence stability in noisy abdominal DWI data.

### Statistical analysis

We applied all six fitting methods to compute pixel-wise maps of IVIM model parameters across the dataset. To assess the impact of b-value sampling on parameter estimation, we performed fitting twice: once using the full set of 13 b-values and once using a reduced set of 7 b-values. As a complementary sensitivity analysis, all methods were also evaluated after excluding the highest b-value of 1200 s/mm$^2$, yielding 12- and 6-b-value variants of the full and reduced protocols, respectively. We conducted all analyses within pancreatic regions of interest (ROIs), specifically the head, body, and tail. For the test-retest experiments, small fixed-size ROIs (12 pixels) were placed in each region to ensure consistent sampling across scans. For the glucose-response experiment, analysis focused on the pancreatic tail. While some studies have reported higher islet density in this region [34], the spatial distribution of islets in the human pancreas remains debated. Recent large-scale and 3D mapping studies have suggested a more homogeneous distribution across the head, body, and tail [35]. These islets of Langerhans, which are highly vascularized and rich in insulin-secreting *β *-cells, can alter local tissue microstructure and capillary perfusion, thereby affecting diffusion- and perfusion-sensitive IVIM parameters. Based on these considerations, we focused on the tail due to its potential functional relevance and its common use in prior studies, while acknowledging regional and inter-individual variability. In this analysis, larger ROIs (mean: 38 pixels) were used to better capture regional signal change while minimizing partial volume effects.

#### Repeatability and agreement analysis

We assessed the repeatability of the estimation of the IVIM parameters using the within-subject coefficient of variation (wCV), calculated for each subject by dividing the standard deviation by the mean of the test–retest measurements for each parameter. We then summarized the distribution of the wCV values between subjects using the median and interquartile range (IQR). We performed paired Wilcoxon signed-rank tests separately for each parameter and b-value configuration to compare repeatability across fitting methods. In addition, we used Bland–Altman analysis to assess agreement between sessions, reporting the mean difference (bias) and the 95% limits of agreement. To evaluate whether IVIM parameter estimates differed significantly across fitting methods, we used the Friedman test separately for each parameter and protocol. In addition, we further compared the wCV between the 13 b-value and 7 b-value protocols for each IVIM parameter and fitting method using a one-sided Wilcoxon signed-rank test, evaluating whether the 13 b-value protocol yielded significantly lower wCV.

#### Fit quality evaluation

To assess the goodness-of-fit for each method, we computed the voxel-wise coefficient of determination ($R^2$), reflecting the proportion of signal variance explained by the model.

#### Neural network stability

We assessed the impact of training variability by training each neural network method 100 times using different random weight initializations. For each subject, we calculated the training coefficient of variation (training CV) across the 100 runs to quantify variability in predicted parameter values due to stochastic training effects. Lower training CV indicated greater model robustness. We also computed the test–retest wCV within each training run to evaluate repeatability at the individual model level.

#### Glucose-response analysis

Glucose-response analysis was performed by comparing pre- and post-glucose IVIM parameter estimates in matched control–T1DM subject pairs. Changes in * f *, * D *, and $D^*$ were assessed across all fitting methods. Pairwise comparisons were used to evaluate relative responses between groups, and model-dependent sensitivity to glucose-induced effects was examined. Due to the small sample size, no formal statistical testing was performed, and results are presented descriptively.

**Fig. 1:**
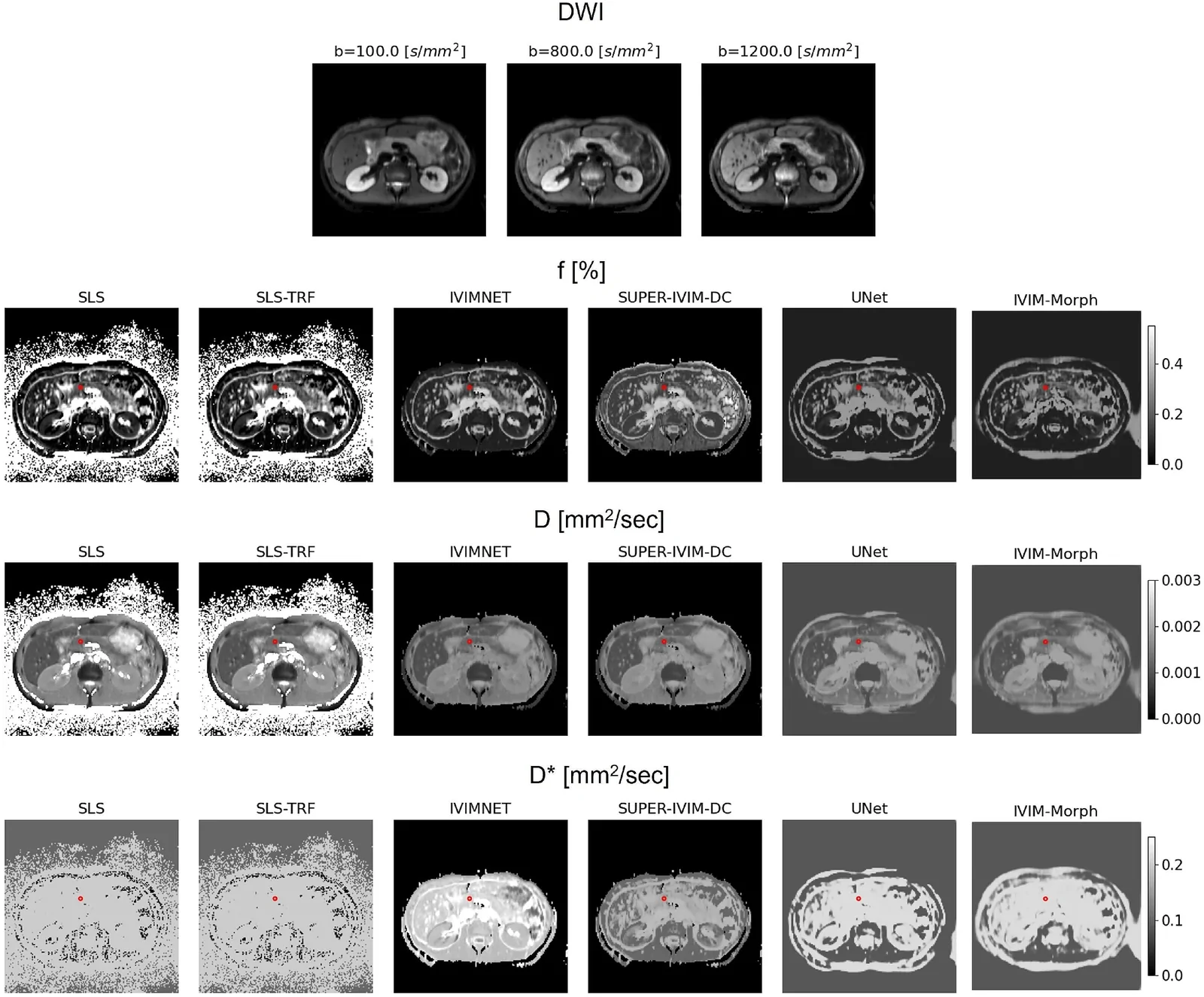
Parameter maps generated by different methods with a red circle highlighting the head of the pancreas. The top row shows DWI images at varying *b*-values, while the rows below display the corresponding *f*-maps, *D*-maps, and $D^*$-maps illustrating differences in parameter estimation across methods

**Fig. 2:**
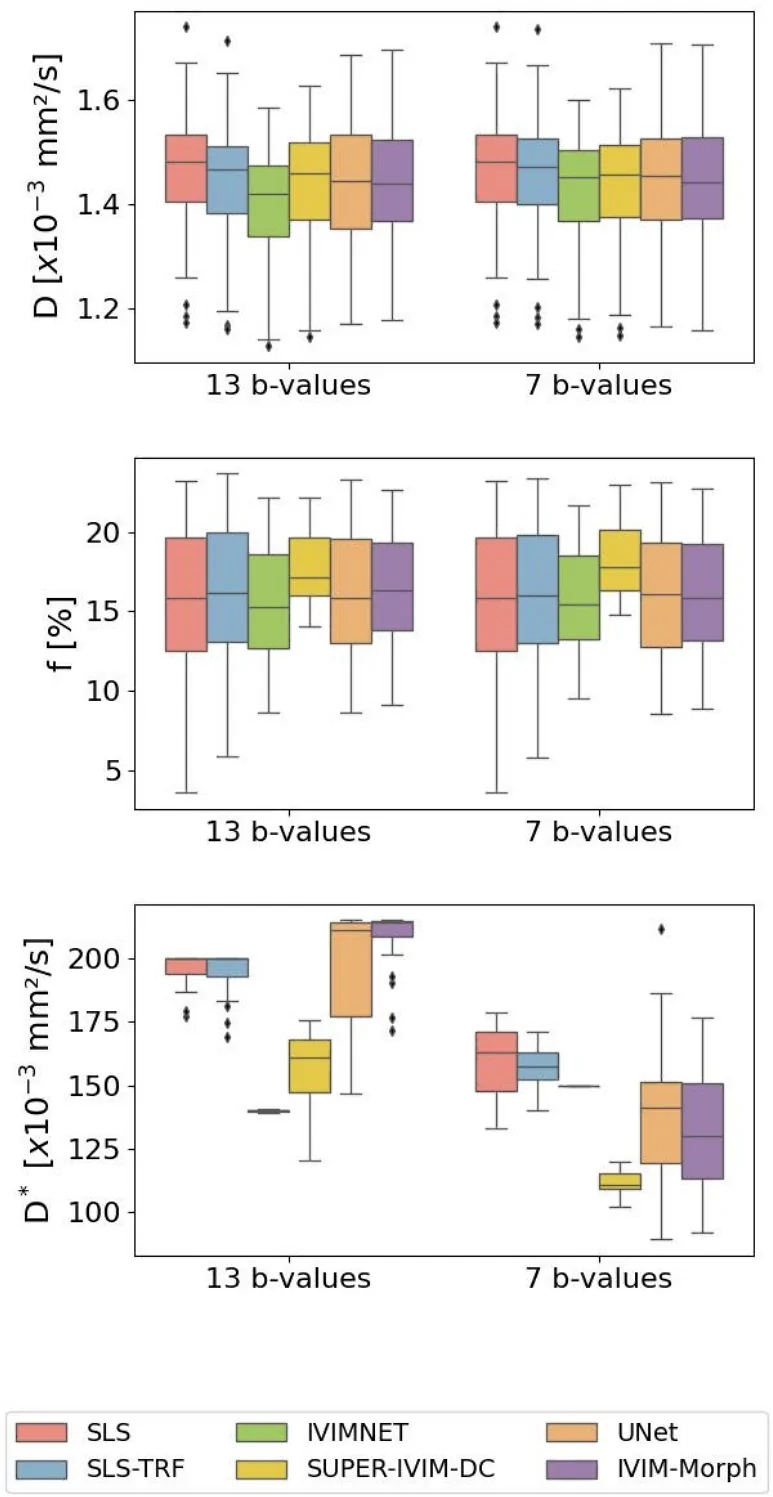
Distribution of IVIM parameters * D *, * f * and $D^*$ estimated using six fitting methods with 7 and 13 b-values

## Results

### Repeatability and agreement

Figure 1 shows representative parametric maps of * f *, * D *, and $D^*$ estimated by the six fitting methods using the 13 b-value protocol. NN-based methods produced smoother and more spatially consistent maps for *f* and *D*. Significant differences were observed between the methods on the $D^*$ maps. IVIM-NET and SUPER-IVIM-DC produced visually smoother $D^*$ maps with fewer apparent outlier regions, whereas classical NLLS, U-Net, and IVIM-Morph frequently showed saturation near the upper bound or a very constrained dynamic range.

Parameter distributions across pancreatic regions are summarized in Fig. 2. For *D*, medians were similar across methods (1.44–1.53 *× * 10$^{-3}$ mm$^2$/s) with narrow IQRs, although classical methods produced more outliers. For *f*, NN-based methods achieved narrower IQRs than classical NLLS, with SUPER-IVIM-DC yielding the highest medians and lowest IQRs. Notably, SLS occasionally generated non-physiological negative *f* values. $D^*$ showed the greatest variability in median and IQR across methods. Friedman tests indicated significant differences between models for all parameters in both protocols (*p<0.05*), indicating that parameter estimates are method-dependent even when median values are similar. These patterns were consistent across all pancreatic segments, as shown in Appendix Figure 7.

The sensitivity analysis excluding the 1200 s/mm$^2$ measurement is shown in Appendix Figure 8. In the conventional segmented fitting methods, exclusion of this b-value resulted in statistically significant but small changes in *D* and *f*, with median changes below 0.4% for *D* and below 0.7% for *f*. For the NN-based methods, significant differences in *D* were observed for IVIMNET, SUPER-IVIM-DC, and IVIM-Morph, but not for UNet. For *f*, significant differences were observed for IVIMNET and SUPER-IVIM-DC, but not for IVIM-Morph or UNet. Changes in $D^*$ were more variable across methods and protocols.

**Fig. 3:**
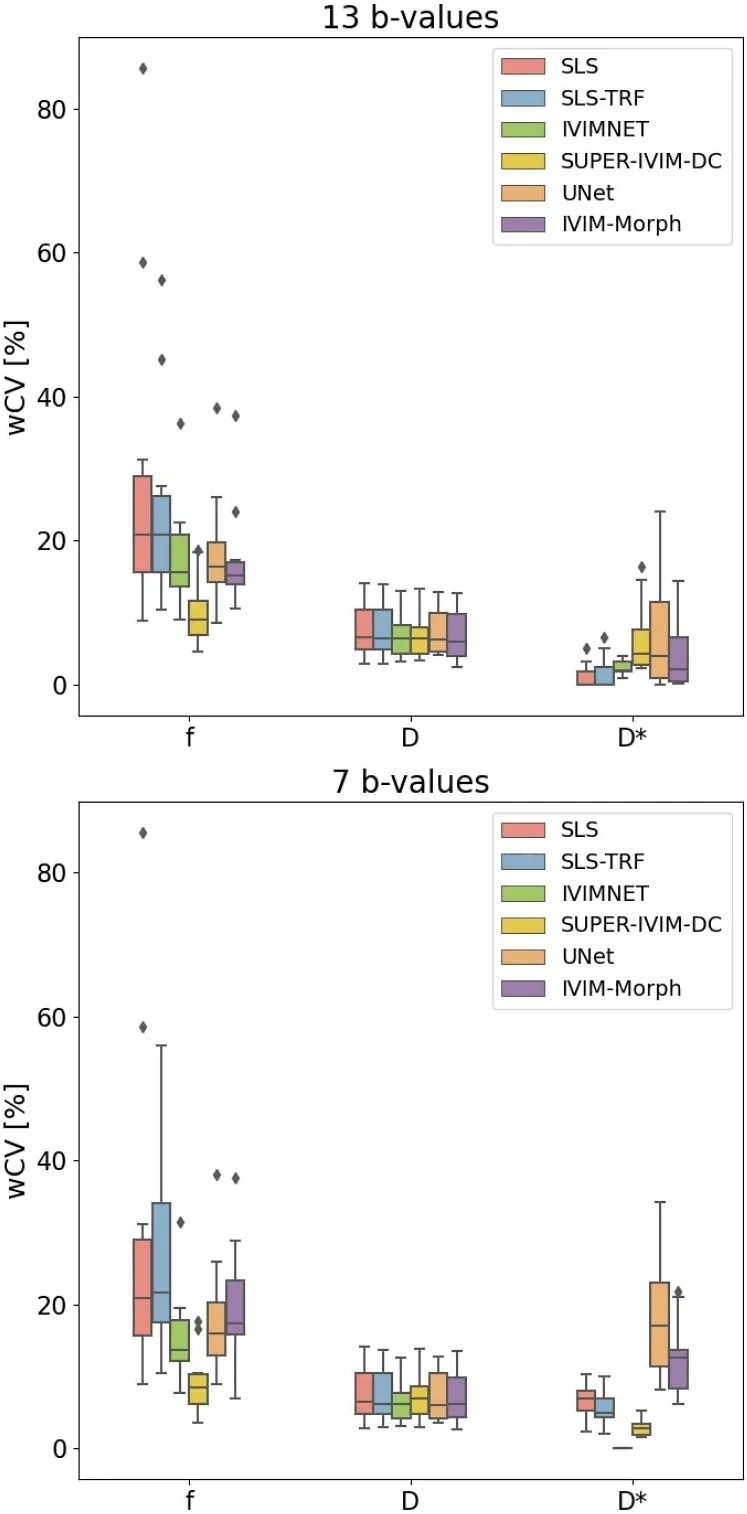
Boxplots of wCV for the mean estimated parameters averaged across the pancreatic head, body, and tail, using 13 b-values (top) and 7 b-values (bottom)

Repeatability metrics (wCV, Fig. 3) demonstrated greater test-retest repeatability of NN-based methods for *f*, with SUPER-IVIM-DC achieving the lowest within-subject variability. (median wCV *≈ *9–10%) compared to classical approaches (*≈ *20%). Two subjects exhibited unusually high wCVs: one primarily in the classical methods and another across all methods, contributing to the wider spread of values observed. For *D*, repeatability was similar across methods (median wCV 6–7%), though NN models tended to show narrower spreads. For $D^*$, performance was inconsistent, with classical methods and IVIMNET performing best under certain protocols, whereas U-Net and IVIM-MORPH exhibited broader variability. One sided pairwise Wilcoxon tests confirmed significantly lower wCVs for *f* in all NN–based methods compared to NLLS-based methods (*p < 0.05*). For *D*, IVIMNET, SUPER-IVIM-DC, and IVIM-Morph showed significantly lower wCV than SLS, while IVIMNET and IVIM-Morph also showed significantly lower wCV than SLS-TRF.

The comparison of the wCV between the 13 b-value and 7 b-value protocols showed that the 7 b-value protocol significantly improved repeatability for *f* in SLS, IVIMNET, and SUPER-IVIM-DC. For *D*, improvements were seen in SLS and IVIMNET, and for $D^*$, in IVIMNET and SUPER-IVIM-DC (*p<0.05*).

**Fig. 4:**
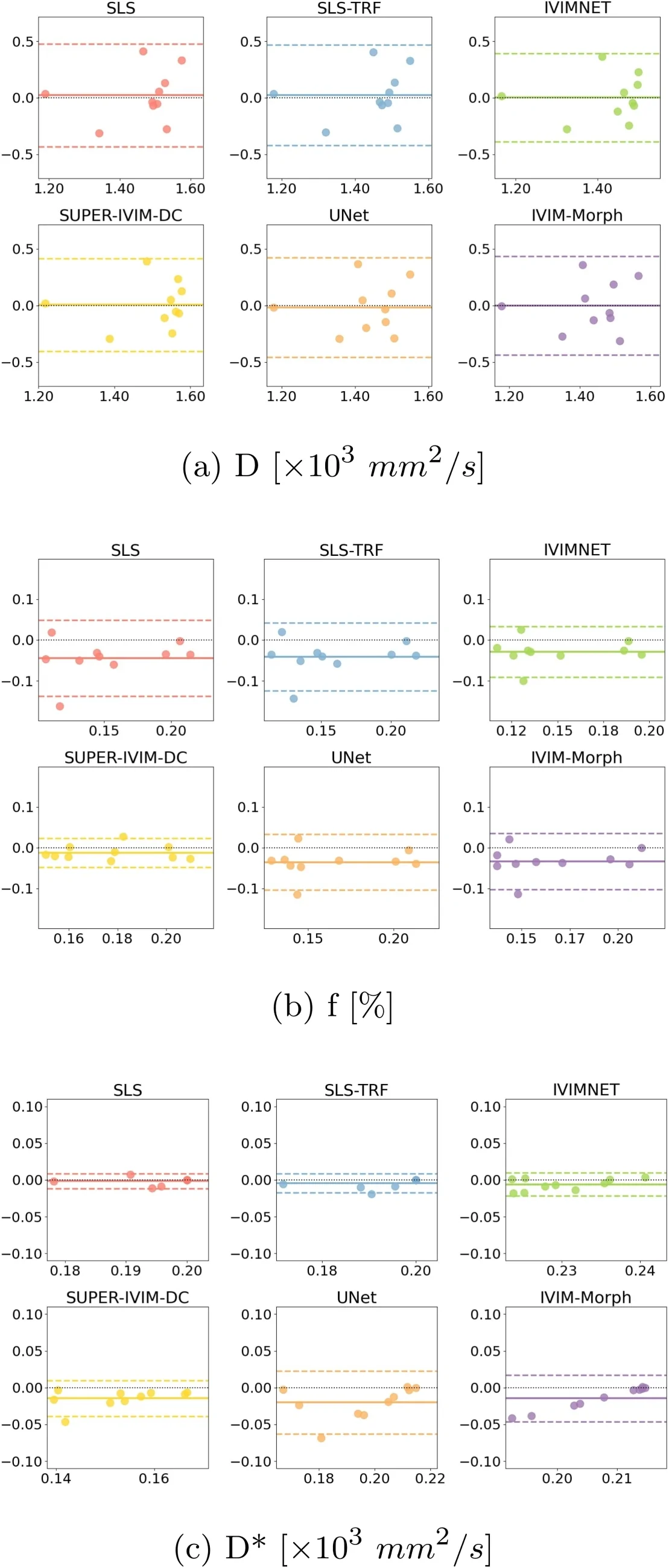
Bland-Altman plots comparing parameter estimates for different models using 13 b-values. The solid line indicates the mean bias, while dashed lines represent the limits of agreement. **a** *D* parameters **b** *f* parameters. **c** $D^*$ parameters

Bland–Altman plots for * f *, * D *, and $D^*$ using 13 b-values are shown in Fig. 4, with 7 b-values in Appendix Figure 9. Bland-Altman analysis was used as a descriptive assessment of test-retest agreement across methods. For *f*, SUPER-IVIM-DC showed the smallest observed bias (–0.012) and narrowest observed limits of agreement (LOA), consistent with its low wCV. Classical methods showed wider observed LOAs, while bias differences were more method-dependent. For * D *, all methods showed low bias but wide LOAs (up to ±0.47), suggesting high variability regardless of method. For $D^*$, performance varied more between methods and protocols. At 13 b-values, classical methods, and IVIM-NET had tighter LOAs and lower bias, while U-Net, IVIM-MORPH, and SUPER-IVIM-DC showed more spread. With 7 b-values, SUPER-IVIM-DC maintained low bias and consistent agreement, whereas IVIM-NET returned zero variability, and both U-Net and IVIM-MORPH displayed greater bias and wider LOAs.

In the two T1D subjects, *f* values were slightly higher than in healthy controls across models (0.18–0.20 vs. 0.12–0.16), whereas *D* values were comparable (1.42–1.55 vs. 1.44–1.53 *× * 10$^{-3}$ mm$^2$/s). As in the healthy cohort, $D^*$ showed marked variability, with U-Net and IVIM-MORPH yielding higher values in T1D. Appendix Figure 10 shows IVIM parameter maps (*f*, *D*, and $D^*$) from a representative T1D subject across all fitting methods, with method-dependent patterns that are similar to those observed in healthy controls. Bland-Altman analysis (Appendix Figure 11) confirmed repeatability in T1D was comparable to healthy controls, with SUPER-IVIM-DC showing the lowest bias for *f* and narrow LOAs, and all methods demonstrating low bias for *D*. For $D^*$, SLS and U-Net yield the tightest LOAs, while other NN-based models exhibited wider but stable ranges.

### Fit Quality

**Fig. 5:**
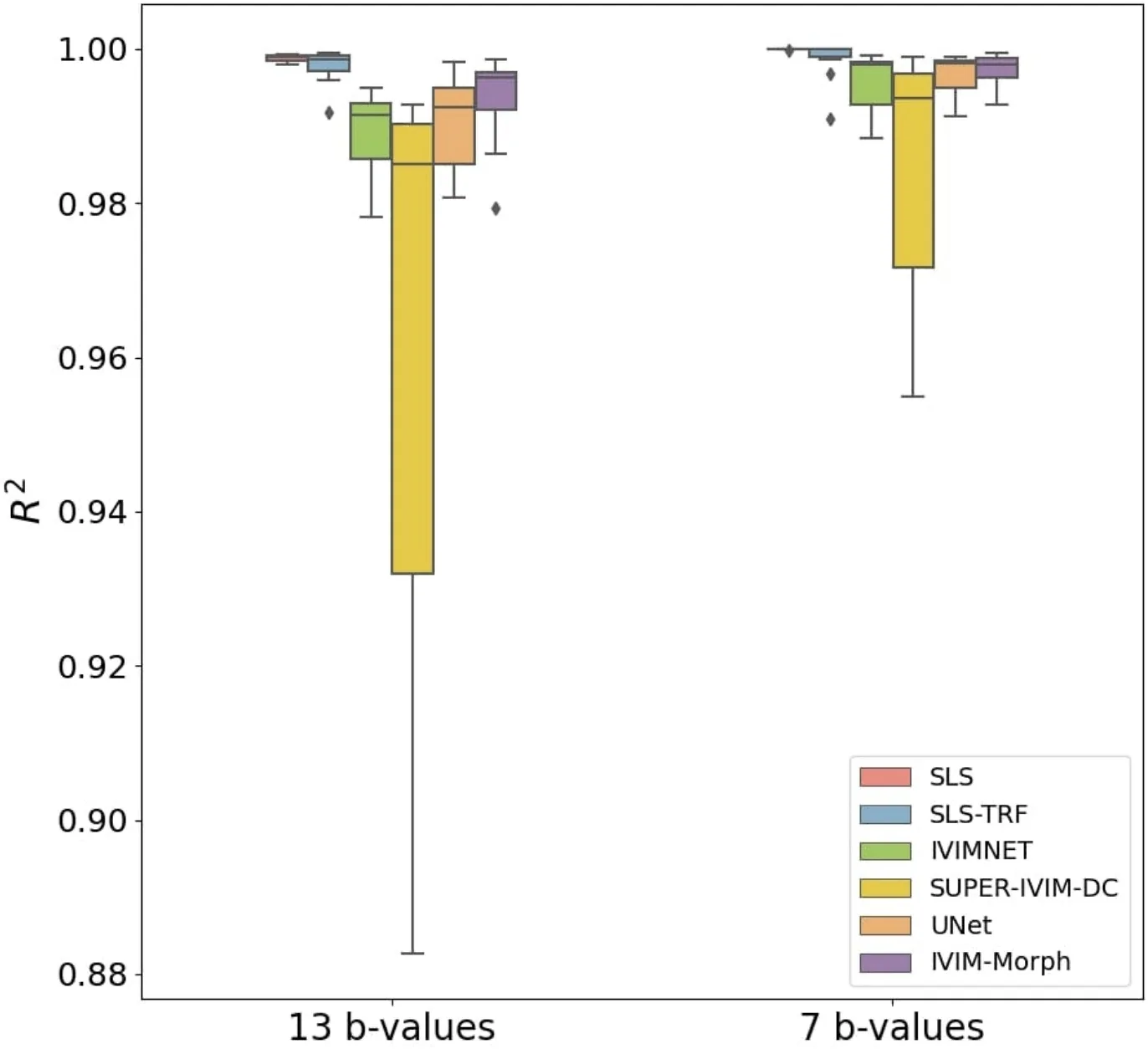
Boxplots of voxel-wise $R^2$ values for each fitting method using 13 and 7 b-values

Figure 5 presents voxel-wise $R^2$ distributions. Classical NLLS methods achieved the highest medians (0.998–0.999) with narrow variability. Among NNs, IVIM-MORPH performed best, with medians close to 0.997 and low variability. U-Net, IVIMNET, and SUPER-IVIM-DC showed slightly lower medians and broader spreads, with SUPER-IVIM-DC exhibiting the widest IQRs. This trend persisted across 100 training repetitions (Appendix Figure 12).

### Neural network stability

We evaluated the CV across 100 independent sessions for each IVIM parameter (Appendix Figure 13). For * f *, SUPER-IVIM-DC consistently demonstrated the lowest median CVs (median CV *≈ *1%). U-Net and IVIM-MORPH exhibited higher variability. For * D *, IVIM-MORPH was most stable at 13 b-values (median CV of 1.17%), while U-Net performed best at 7 b-values (0.87%). For $D^*$, SUPER-IVIM-DC maintained low CVs across protocols, IVIM-NET showed zero variability at 7 b-values but fluctuated more at 13 b-values, and both U-Net and IVIM-MORPH showed the greatest variability.

### Glucose-response

**Fig. 6:**
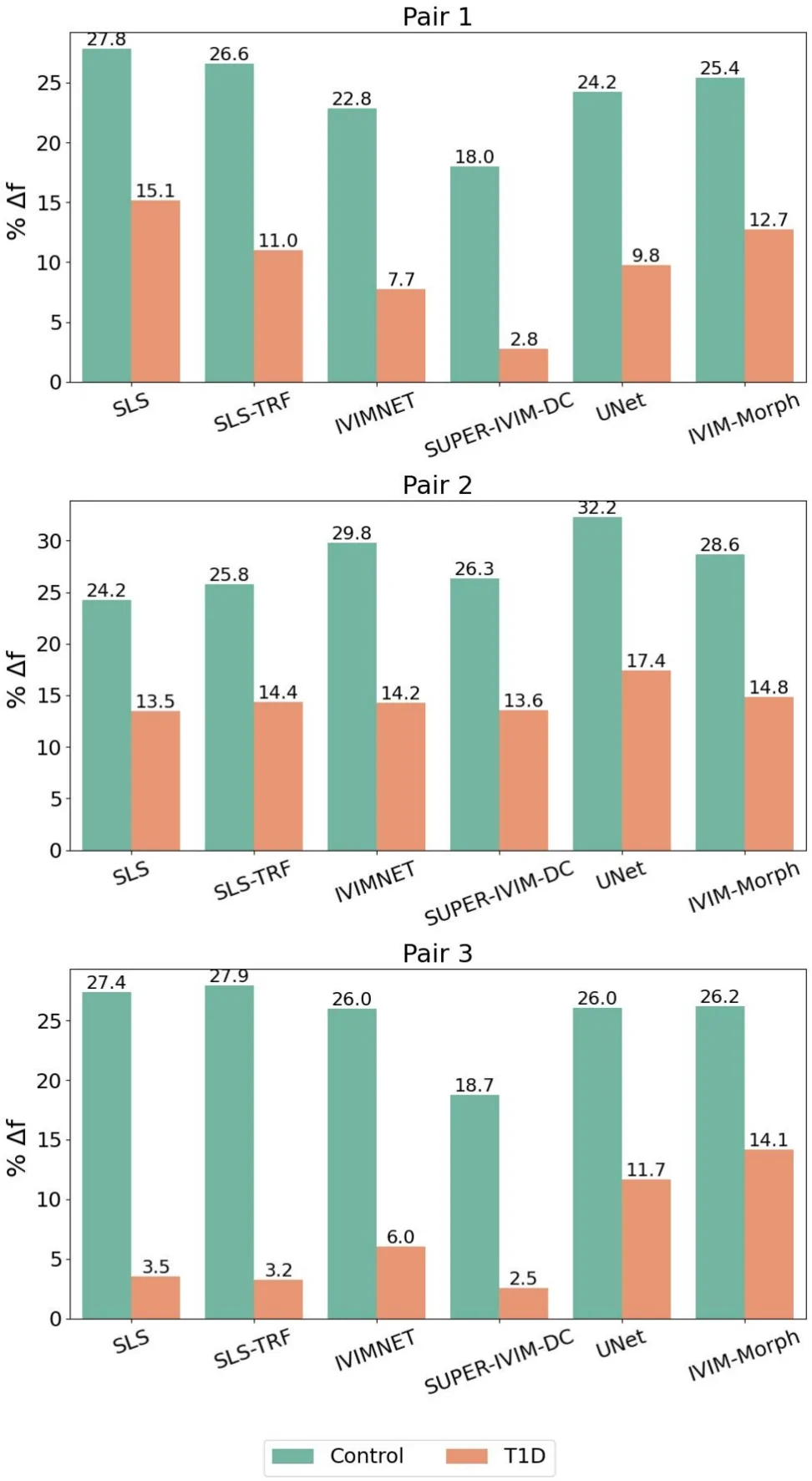
Percent change in *f* parameter before and after glucose intake across models in the pancreatic tail. Each row corresponds to one age- and sex-matched control–T1D pair

All models detected glucose-induced changes in *f* (Fig. 6). Baseline *f* values ranged from 13.4–33.4% in T1DM and 13.2–17.2% in controls, with post-glucose values spanning 14.2–37.4% and 17.1–21.6%, respectively. In pairwise comparisons, control subjects generally exhibited larger increases than T1DM. In pairs 1 and 2, SLS-TRF showed the strongest separation, whereas in pair 3, IVIMNET provided the clearest discrimination. Across pairs, IVIM-MORPH, U-Net, and SUPER-IVIM-DC produced consistent but more moderate changes. For *D*, responses were heterogeneous. Controls showed mixed increases and decreases, while only one T1DM subject exhibited a post-glucose increase (Appendix Figure 14). For $D^*$, no consistent patterns were observed across models or pairs (Appendix Figure 15), highlighting limited reliability of this parameter for detecting glucose-related changes.

## Discussion

IVIM MRI is a valuable tool for probing tissue microstructure and perfusion, but pancreatic applications remain challenging due to motion artifacts and tissue heterogeneity. Reliable and repeatable parameter estimation is therefore essential. In this study, we systematically compared four NN-based IVIM fitting methods with two classical NLLS approaches in the pancreas, focusing on repeatability, agreement, stability, and acquisition efficiency.

Parameter values were broadly consistent with previous pancreatic IVIM reports [11], but statistical testing confirmed significant inter-model differences, showing that estimates are method-dependent even when medians appear similar. The wider voxel-wise distributions observed with classical methods may partly reflect true spatial heterogeneity in pancreatic perfusion, rather than fitting instability alone. However, the presence of outliers and occasional non-physical estimates suggests that at least part of this variability may also be influenced by noise, motion, or fitting instability. Thus, classical methods may preserve greater apparent voxel-wise heterogeneity, whereas NN-based methods showed more repeatable estimates across repeated scans in this dataset. Non-zero parameter values were also observed outside the pancreas, including in subcutaneous fat. These values are not physiologically interpretable and likely reflect the low underlying DWI signal in fat and residual motion around the pancreas, both of which increase susceptibility to noise-floor effects and can destabilize parameter estimation.

NN-based approaches generally showed improved test-retest repeatability relative to NLLS for the perfusion fraction *f*, as reflected by narrower interquartile ranges and lower wCVs. These improvements align with prior work showing the value of deep learning in abdominal imaging under noisy, motion-prone conditions [15, 24]. Among the NN methods, SUPER-IVIM-DC provided the most repeatable *f* estimates, with the lowest wCV and smallest descriptive Bland-Altman bias, making it particularly relevant when perfusion-sensitive changes are clinically relevant. IVIM-MORPH, in contrast, provided the most balanced performance, with consistently low variability in *D* and stable training behavior across both protocols, suggesting suitability for routine use when all IVIM parameters are of interest. Variability in $D^*$ remained substantial across all models and protocols, consistent with the known ill-posed nature of this parameter [36]. Classical methods and IVIM-NET showed relatively low wCV under certain protocols, whereas U-Net, IVIM-MORPH, and SUPER-IVIM-DC showed broader variability under the reduced 7-b-value protocol. However, because the pseudo-diffusion component is encoded primarily in low-b measurements that are highly sensitive to noise, its estimation remained inherently unstable. Therefore, future work should consider fixing $D^*$ to a physiologically plausible a priori value, as several studies have shown that constraining this parameter can stabilize the IVIM model and improve the robustness of *D* and *f* estimates[37].

The $R^2$ analysis provides complementary information to the repeatability metrics. NLLS achieved the highest median $R^2$, consistent with its direct voxel-wise optimization of the signal decay. In comparison, NN-based methods showed slightly lower median $R^2$ values, while remaining high overall, and some methods showed broader $R^2$ distributions, indicating greater variability in voxel-wise signal reconstruction. These results indicate that classical fitting provided the closest voxel-wise agreement with the measured diffusion signal, whereas NN-based methods provided more repeatable estimates of the perfusion fraction *f* across repeated scans. Together, these findings show that signal-fitting fidelity and parameter repeatability capture distinct aspects of IVIM model performance and should both be considered when evaluating fitting methods.

An important finding of this study is that reducing the protocol to seven strategically chosen b-values yielded repeatability comparable to, and in some cases better than, the full 13 b-value set. SLS, IVIM-NET, and SUPER-IVIM-DC benefited from the shorter protocol, likely because these methods are sensitive to motion and noise artifacts at very low b-values, which were reduced in the optimized protocol. Methods with inherent motion-robust design (IVIM-MORPH) or lower sensitivity (SLS-TRF, U-Net) performed similarly under both protocols. These findings support prior optimization studies emphasizing the placement rather than the number of b-values [29]. The impact of protocol reduction was also reflected in model fit quality. The full 13 b-value protocol produced a wider spread of $R^2$ values, likely due to its inclusion of multiple very low b-values, which enhance perfusion sensitivity but may also increase sensitivity to respiratory motion, flow-related effects, and instability in perfusion-related parameter estimation. By omitting several of these points, the optimized 7 b-value protocol produced narrower $R^2$ distributions and more stable parameter estimates. Clinically, the shortened protocol also reduces scan time to under five minutes, improving patient comfort and workflow efficiency without sacrificing reliability. In addition to the reduction of very low b-values, the influence of high b-values was also evaluated. At high b-values, non-Gaussian diffusion (kurtosis effects) may affect IVIM estimates. Excluding 1200 s/mm$^2$ led to small but statistically significant changes in *D* and *f*, with more variable effects on $D^*$, indicating a limited, method-dependent impact on parameter estimates.

Training stability analysis across 100 independent sessions confirmed that NN-based methods maintain consistent performance across repeated trainings, addressing a critical concern for clinical translation. SUPER-IVIM-DC consistently produced low CVs and narrow IQRs for *f*, while IVIM-NET achieved the lowest variability for *D* across both protocols. Although classical NLLS methods achieved high $R^2$ values, they showed wider spreads in *f*, which may reduce sensitivity to small perfusion-related changes.

Preliminary results in two T1D subjects showed slightly higher median *f* values compared to healthy controls, while *D* values were comparable and $D^*$ remained variable across models. Test–retest results in these cases appeared broadly similar to healthy subjects, but the very small sample precludes firm conclusions. The glucose-response experiment demonstrated consistent increases in *f* after glucose intake, with control subjects generally showing larger changes than T1DM. This trend is physiologically plausible, as glucose ingestion increases pancreatic perfusion through enhanced microvascular recruitment, an effect that may be blunted in T1DM due to impaired vascular reactivity and reduced islet microcirculation [10]. The degree of separation was method-dependent: SLS-TRF and IVIMNET showed the clearest group differences, whereas U-Net, IVIM-MORPH, and SUPER-IVIM-DC produced more moderate but consistent responses. Thus, while NN-based methods were able to capture glucose-related changes, it remains unclear whether they provide superior discrimination compared to classical approaches. Larger studies are required to establish whether the more conservative responses observed in NNs reflect greater robustness or reduced sensitivity.

Several limitations should be acknowledged. First, the study cohort was small and dominated by healthy volunteers, with only limited T1D cases. Although this sample allowed us to evaluate repeatability and compare the fitting methods, larger and more diverse patient cohorts will be required to establish clinical applicability. Second, data were acquired on a single scanner platform, which limits the assessment of cross-scanner robustness. Validation across multiple scanners and centers will therefore be an important next step to determine generalizability. Third, while we reported $R^2$ as a fit-quality metric, it can be misleading in exponential models [38]; complementary metrics such as residual analysis or uncertainty estimates should be included in future studies. In addition, although the NN-based methods showed improved repeatability, the observed variability in absolute estimates and percent changes across architectures suggests that additional validation is needed before drawing firm conclusions regarding their absolute accuracy. Finally, while further systematic optimization of b-value selection may yield even more efficient protocols, in this study, we applied the same full and reduced b-value sets across all methods to ensure a fair comparison. This choice allowed us to attribute performance differences to the fitting strategy itself, but may have limited the opportunity to explore method-specific acquisition optimization.

## Conclusion

This work demonstrates that NN-based IVIM fitting methods improved test–retest repeatability of the perfusion fraction *f* compared with classical NLLS approaches in pancreatic imaging, while performance for *D* and $D^*$ was more method- and protocol-dependent. Among the evaluated networks, SUPER-IVIM-DC showed the lowest wCV and smallest descriptive Bland–Altman bias for *f*, particularly when combined with the optimized 7 b-value protocol, and may therefore be useful when repeatable perfusion-sensitive biomarkers and shorter scan times are prioritized. IVIM-MORPH showed balanced performance across parameters and stable behavior for both the 7 and 13 b-value protocols, making it a practical option when joint assessment of *D*, *f*, and $D^*$ is required. Reducing the acquisition to seven carefully selected b-values maintained or improved repeatability in several method–parameter combinations while shortening scan time, supporting further evaluation of this protocol in clinical workflows. Preliminary results in T1D further highlight the potential of IVIM analysis to detect physiologically meaningful changes, supporting further investigation of NN-based approaches in broader clinical studies.

## Figures and Tables

**Fig. 7:**
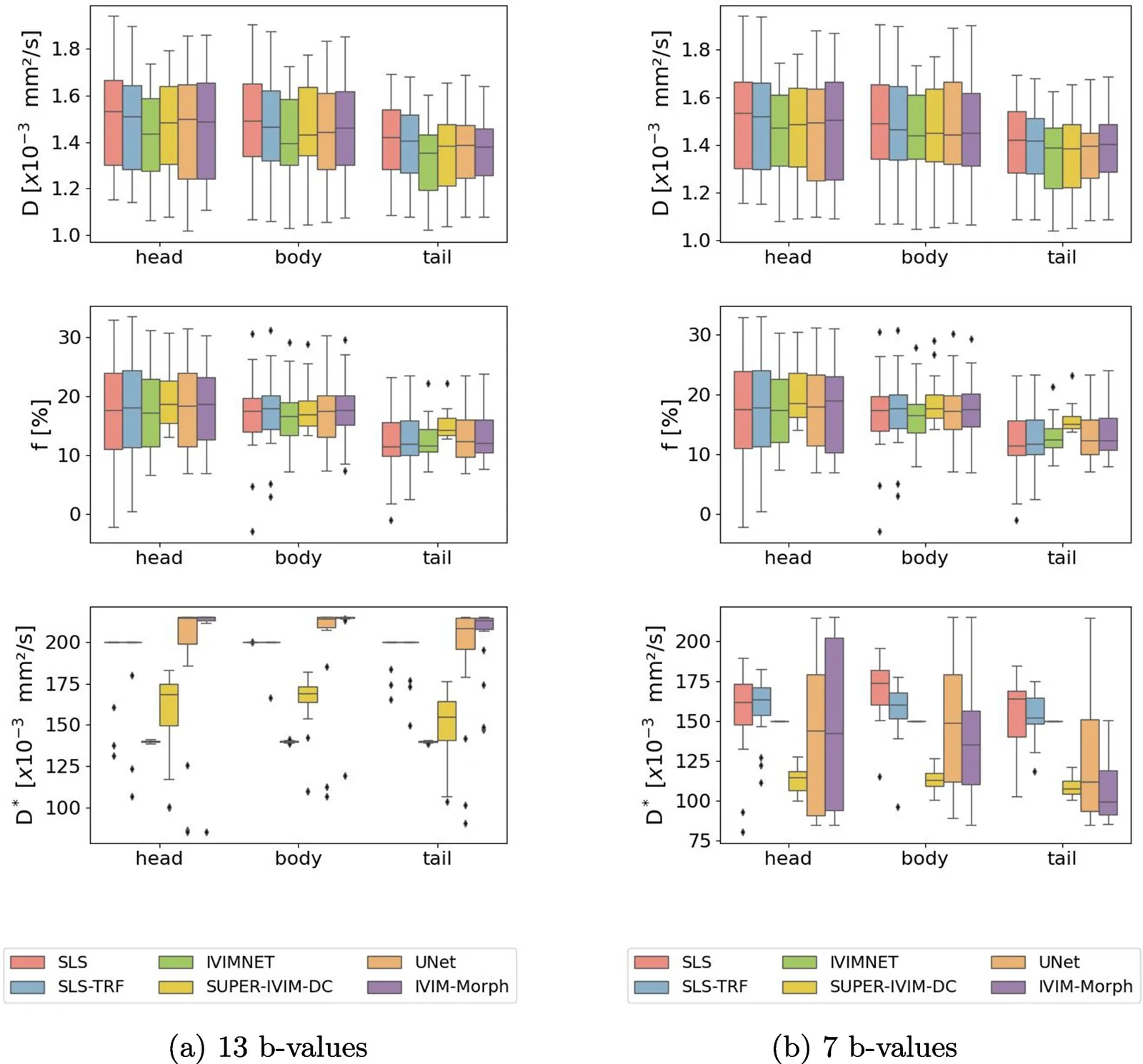
Boxplots of wCV for estimated parameters across the pancreatic head, body, and tail, using **a** 13 b-values and **b** 7 b-values

**Fig. 8:**
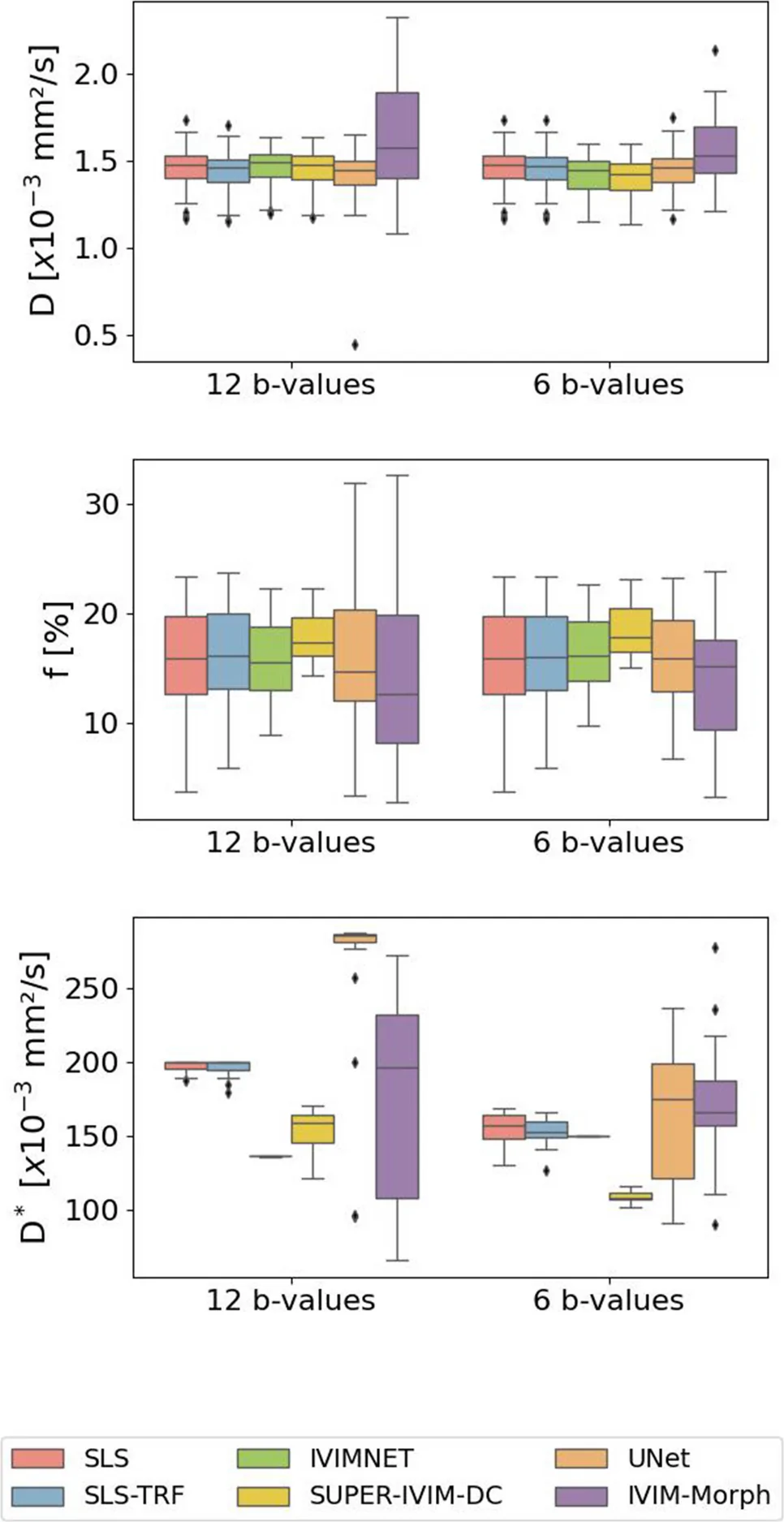
Sensitivity analysis excluding the highest b-value of 1200 s/mm$^2$. Parameter distributions are shown after removal of this b-value for the full protocol (12 b-values) and the reduced protocol (6 b-values)

**Fig. 9:**
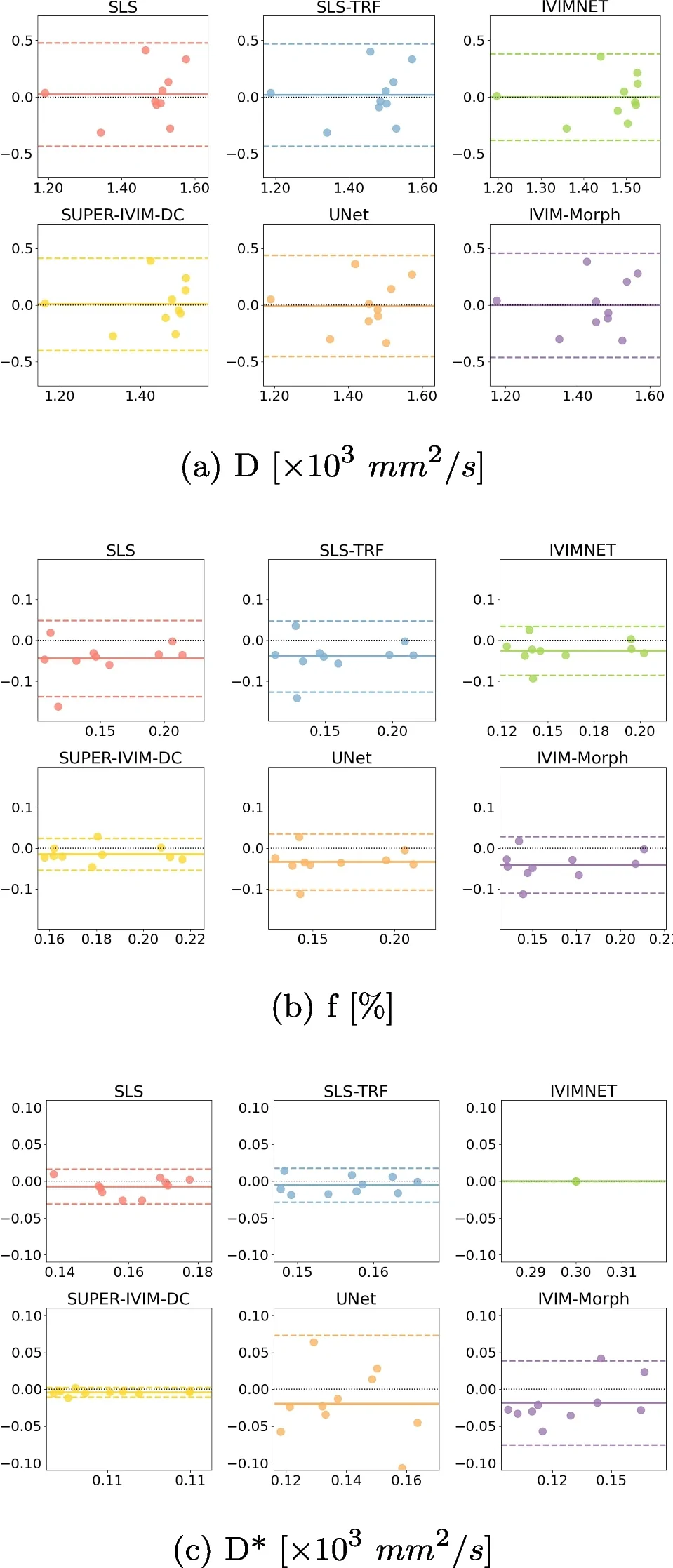
Bland-Altman plot comparing parameter estimates for different models using 7 b-values. The solid black line indicates the mean bias, while dashed lines represent the limits of agreement. **a** D parameter **b** f parameter **c** D* parameter

**Fig. 10:**
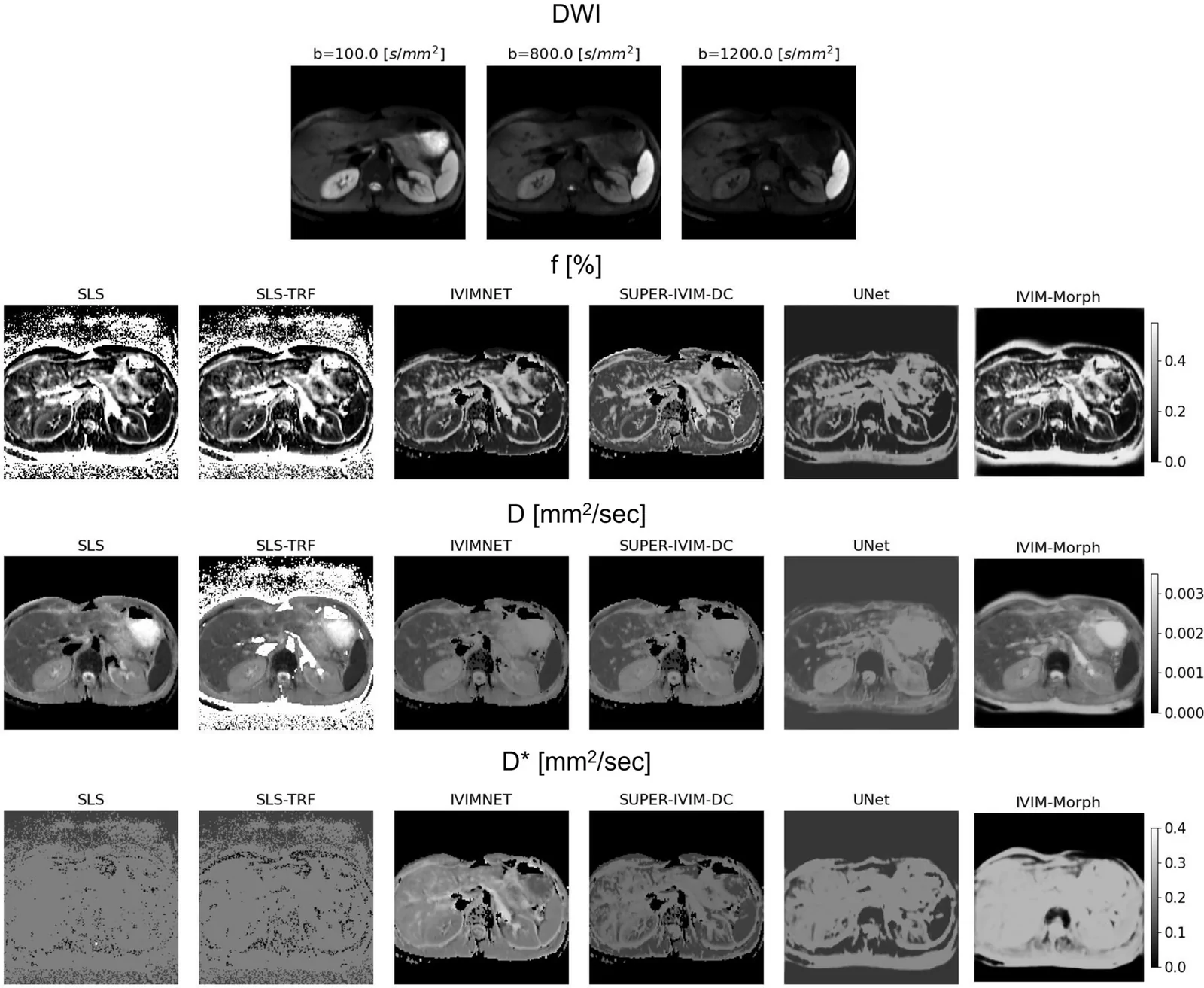
Representative parameter maps of T1D subject generated by different methods. The top row shows DWI images at varying *b*-values, while the rows below display the corresponding *f*-maps, *D*-maps, and $D^*$-maps illustrating differences in parameter estimation across methods

**Fig. 11:**
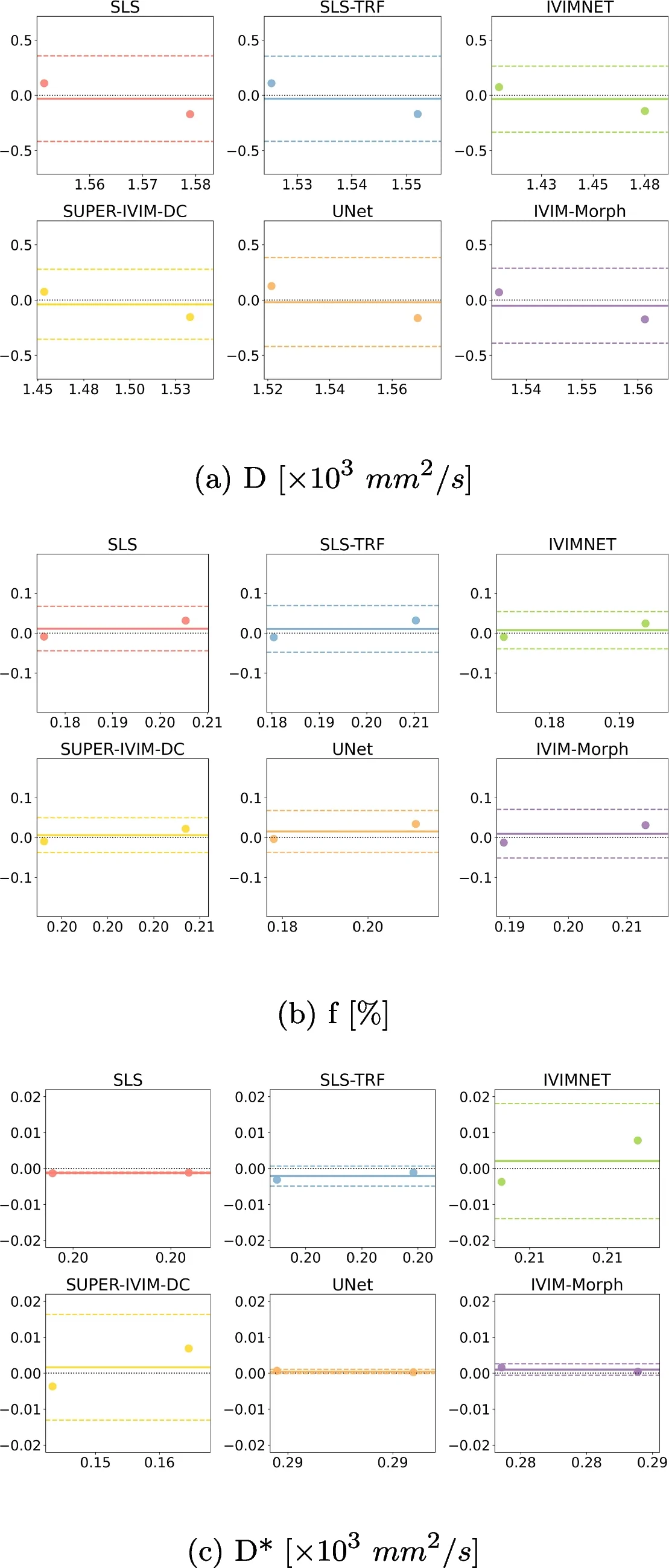
Bland-Altman plots comparing parameter estimates for different models using 13 b-values for the T1D cases. The solid line indicates the mean bias, while dashed lines represent the limits of agreement. **a** *D* parameters **b** *f* parameters. **c** $D^*$ parameters

**Fig. 12:**
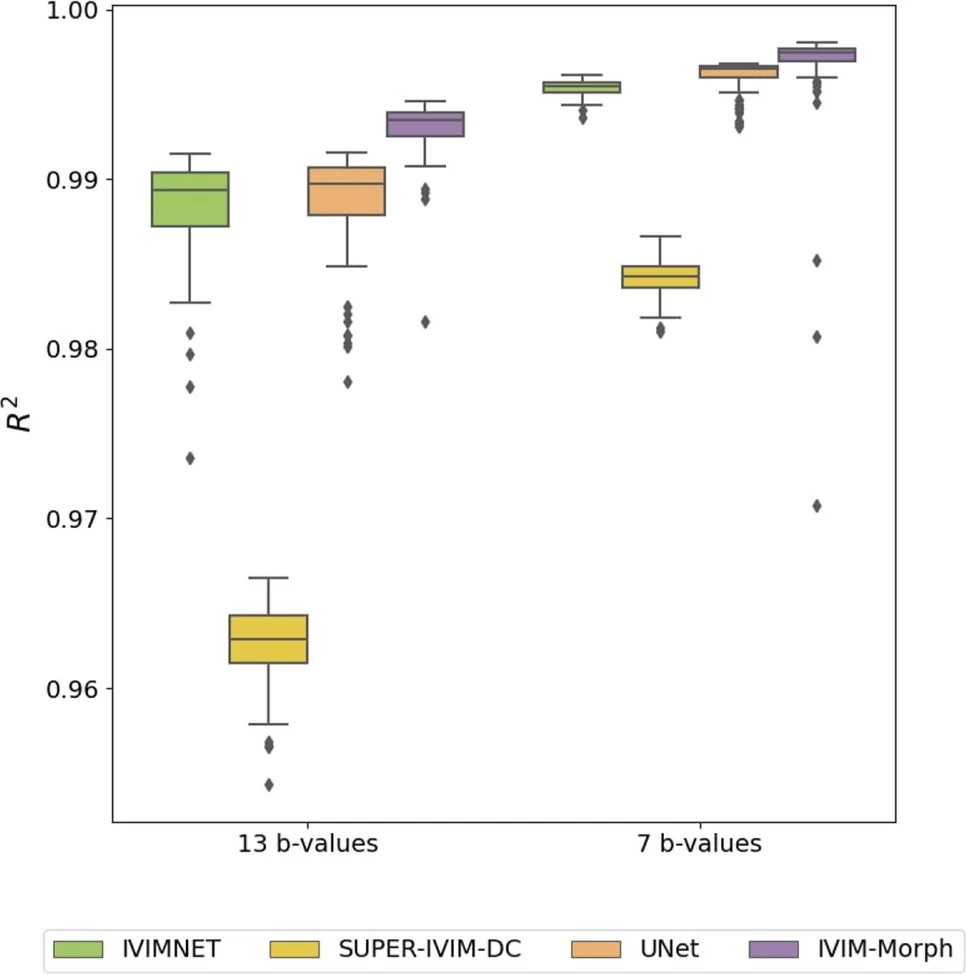
Boxplots $R^2$, computed within each of the 100 independent training sessions

**Fig. 13:**
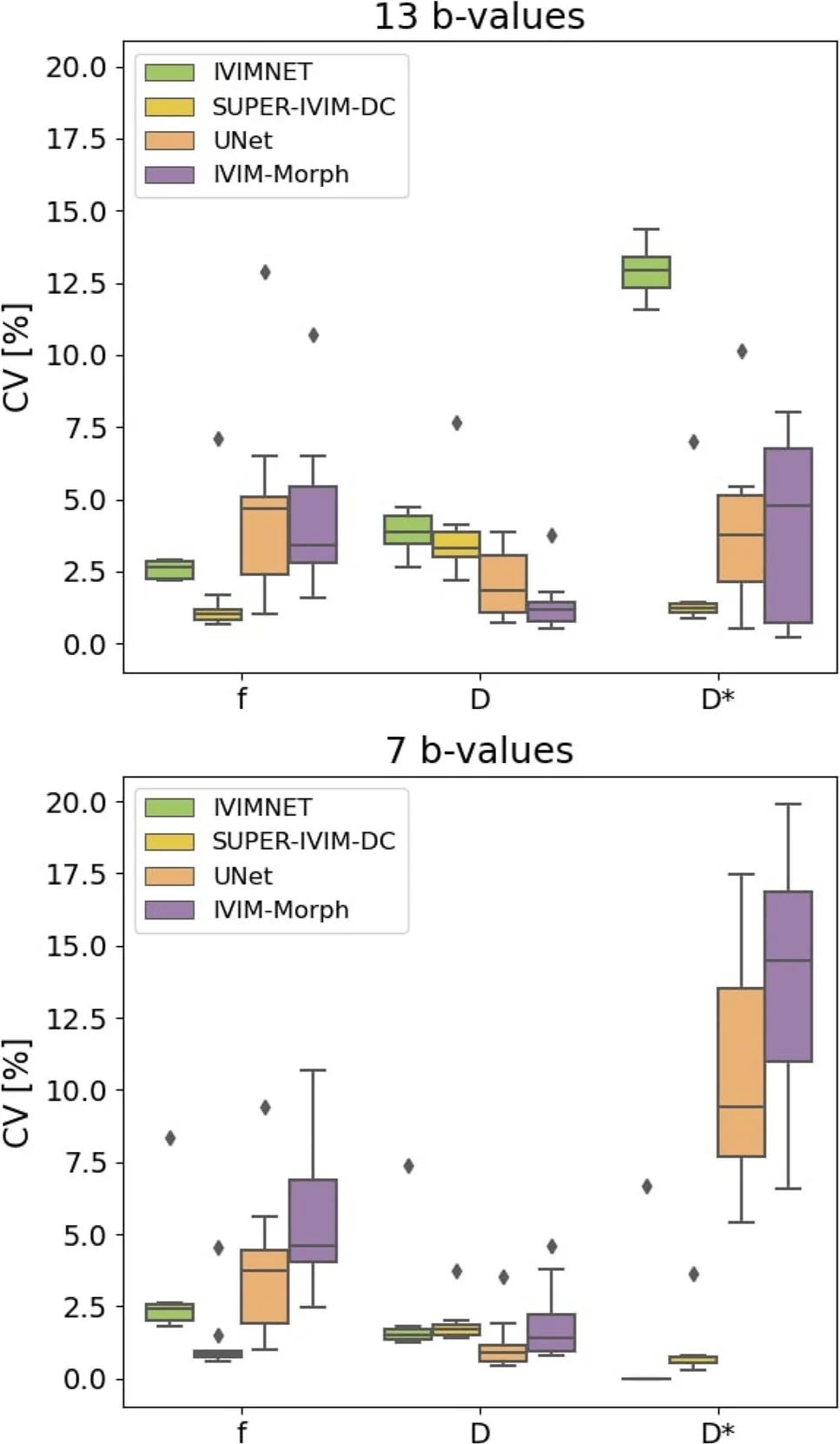
Boxplots of CV across 100 training sessions for each NN-based method, calculated per subject within the test data ROI

**Fig. 14:**
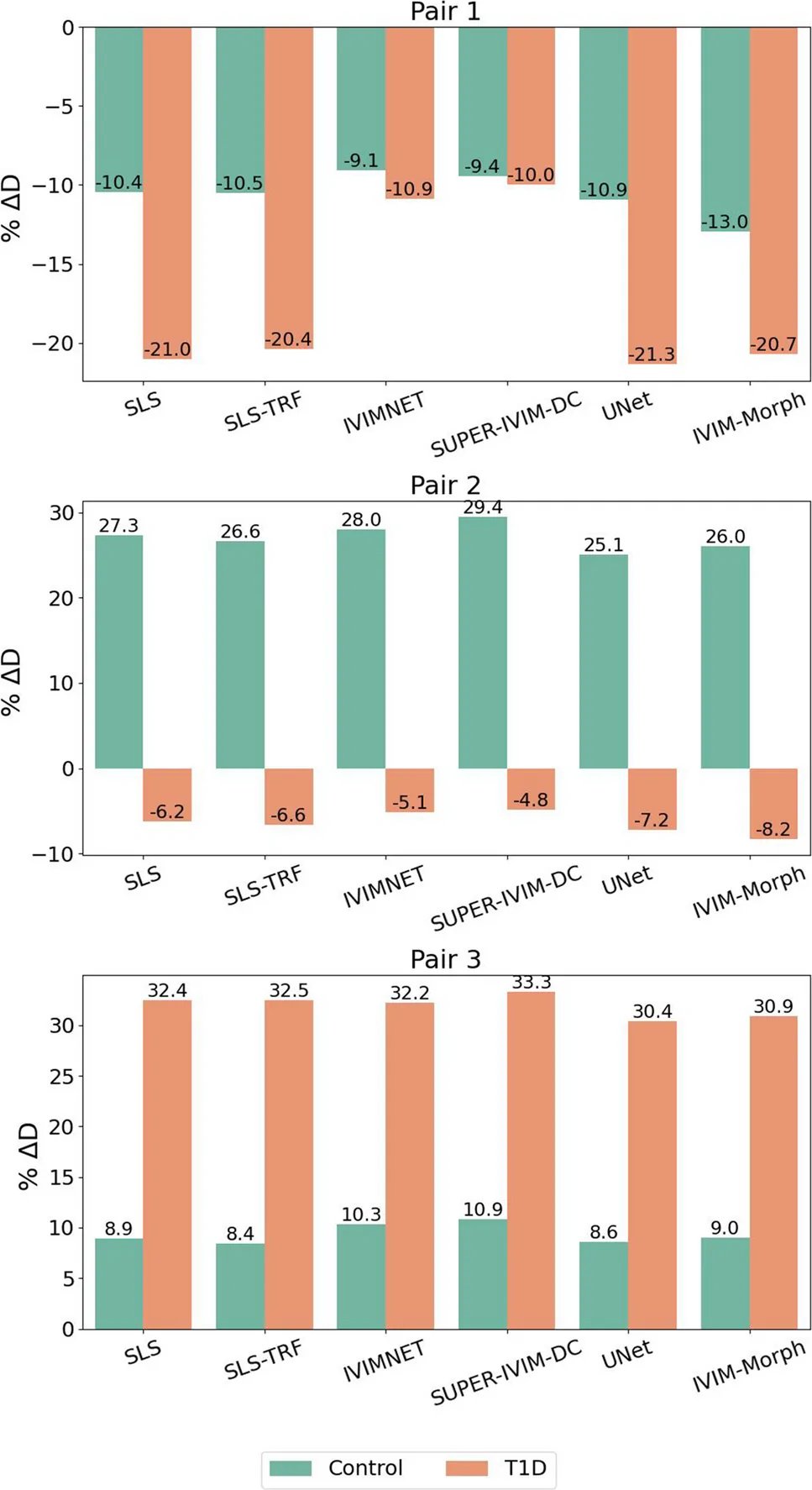
Percent change in *D* parameter before and after glucose intake across models in the pancreatic tail. Each row corresponds to one age- and sex-matched control–T1D pair

**Fig. 15:**
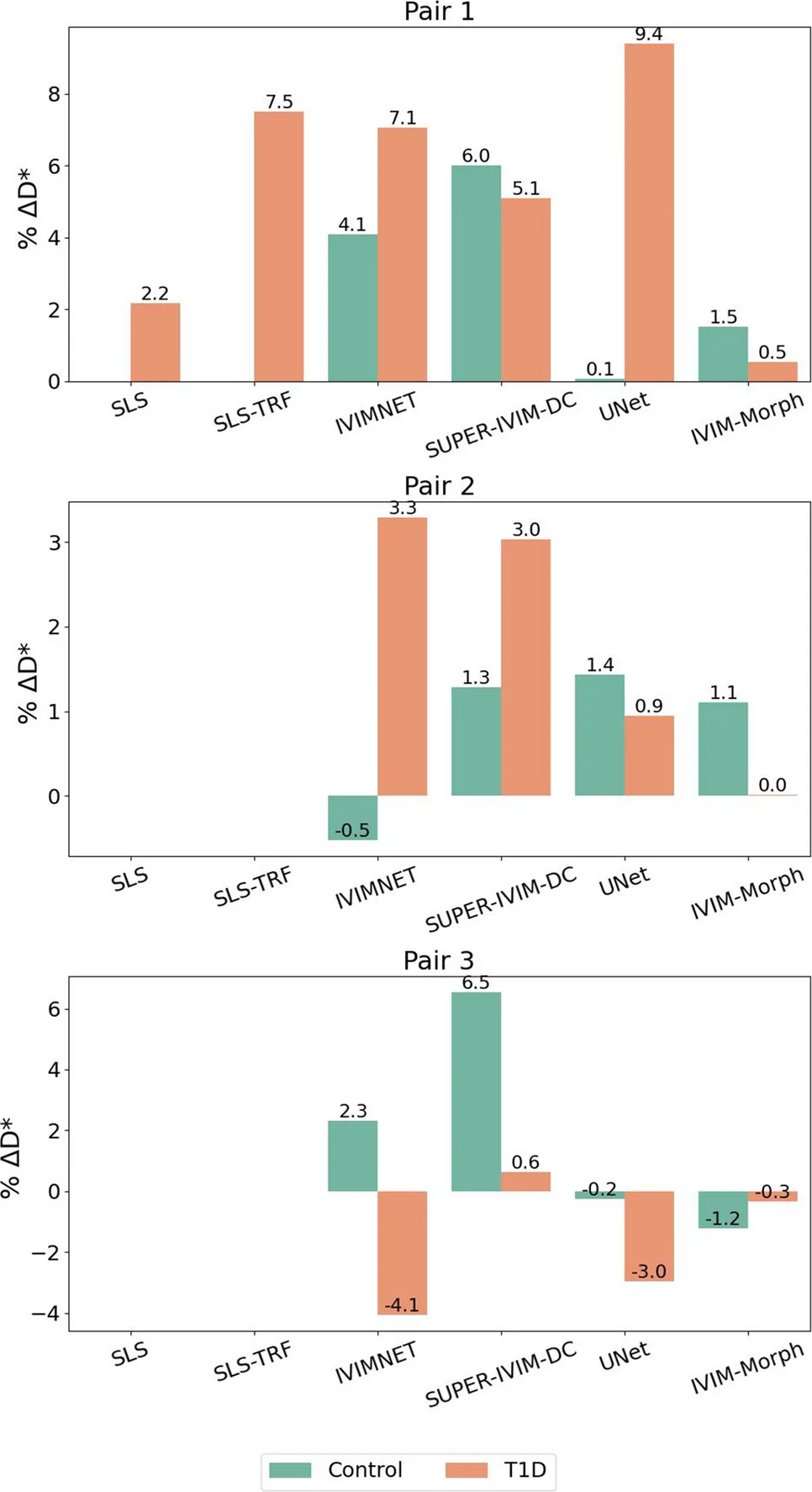
Percent change in $D^*$ parameter before and after glucose intake across models in the pancreatic tail. Each row corresponds to one age- and sex-matched control–T1D pair

## Declarations

### Generative AI

During the preparation of this work, the author(s) used ChatGPT in order to improve readability. After using this tool/service, the author(s) reviewed and edited the content as needed and take(s) full responsibility for the content of the publication.
